# Incipient sympatric speciation in Midas cichlid fish from the youngest and one of the smallest crater lakes in Nicaragua due to differential use of the benthic and limnetic habitats?

**DOI:** 10.1002/ece3.2287

**Published:** 2016-07-01

**Authors:** Andreas F. Kautt, Gonzalo Machado‐Schiaffino, Julian Torres‐Dowdall, Axel Meyer

**Affiliations:** ^1^ Department of BiologyUniversity of KonstanzUniversitätsstrasse 1078457KonstanzGermany

**Keywords:** Demographic inference, habitat isolation, individual specialization, phenotypic plasticity, RAD‐seq, stable isotopes

## Abstract

Understanding how speciation can occur without geographic isolation remains a central objective in evolutionary biology. Generally, some form of disruptive selection and assortative mating are necessary for sympatric speciation to occur. Disruptive selection can arise from intraspecific competition for resources. If this competition leads to the differential use of habitats and variation in relevant traits is genetically determined, then assortative mating can be an automatic consequence (i.e., habitat isolation). In this study, we caught Midas cichlid fish from the limnetic (middle of the lake) and benthic (shore) habitats of Crater Lake Asososca Managua to test whether some of the necessary conditions for sympatric speciation due to intraspecific competition and habitat isolation are given. Lake As. Managua is very small (<900 m in diameter), extremely young (maximally 1245 years of age), and completely isolated. It is inhabited by, probably, only a single endemic species of Midas cichlids, *Amphilophus tolteca*. We found that fish from the limnetic habitat were more elongated than fish collected from the benthic habitat, as would be predicted from ecomorphological considerations. Stable isotope analyses confirmed that the former also exhibit a more limnetic lifestyle than the latter. Furthermore, split‐brood design experiments in the laboratory suggest that phenotypic plasticity is unlikely to explain much of the observed differences in body elongation that we observed in the field. Yet, neutral markers (microsatellites) did not reveal any genetic clustering in the population. Interestingly, demographic inferences based on RAD‐seq data suggest that the apparent lack of genetic differentiation at neutral markers could simply be due to a lack of time, as intraspecific competition may only have begun a few hundred generations ago.

## Introduction

Empirical studies of the conditions that may allow for or constrain the process of speciation with gene flow and especially its most extreme form, sympatric speciation, are essential to test theoretical predictions (Bolnick 2011; Martin 2013; Meyer and Kautt 2014; Comeault et al. 2015). Generally, theory has revealed that ecological sympatric speciation requires some form of disruptive selection and assortative mating to overcome the homogenizing process of gene flow and recombination (Felsenstein 1981; Gavrilets 2003, 2004). Disruptive selection can arise from intraspecific competition, which may be apparent by individual specialization to certain food resources (Martin and Pfennig 2009). The conditions for sympatric speciation are very restricted (Gavrilets 2005), and only if the strength of disruptive selection and the probability of assortative mating are sufficiently strong may sympatric speciation occur (Udovic 1980). Moreover, if there are costs associated with choosing a mate, the likelihood of sympatric speciation is strongly reduced (Matessi et al. 2001). On the other hand, if only few loci of large effect are underlying ecologically relevant traits and those conferring assortative mating and both are closely genetically linked (e.g., in close physical proximity or located on an inversion), sympatric speciation is facilitated as their association will be less frequently broken down by recombination (Trickett and Butlin 1994; Rieseberg 2001). In the most extreme case, traits under selection and those that confer assortative mating are one and the same (Gavrilets 2004). Consequently, if individual specialization leads automatically to assortative mating due to the spatial segregation of mating pools (habitat isolation) or influences the propensity of individuals to mate with others of a similar phenotype (mate preference), population divergence in sympatry is greatly facilitated (Gavrilets 2004; Servedio et al. 2011; Smadja and Butlin 2011). Importantly, the variation in ecologically relevant traits and thus individual specialization has to be genetically determined and not due to phenotypic plasticity: While adaptive phenotypic plasticity might increase the potential for ecological speciation in the long term by allowing for the colonization of new environments and population persistence, it will usually impede population divergence in sympatry (Bolnick 2011; Thibert‐Plante and Hendry 2011).

Thus, some of the necessary (but not sufficient) conditions for sympatric speciation due to intraspecific competition for resources and habitat isolation are as follows: (1) individuals differ in ecomorphological traits, (2) their ecomorphological characteristics match, and individuals differentially use, distinct habitats, and (3) variation in ecologically relevant traits is genetically determined and not due to phenotypic plasticity. At the genomic level, differentiation in this process is initially expected to be only reflected at the few loci under disruptive selection, whereas genome‐wide differentiation will be virtually absent and only build up with time after gene flow has ceased (Wu 2001; Nosil et al. 2009a; Feder et al. 2012).

Competition for resources is thought to be the main driver of divergence between the ground‐associated (benthic) and open‐water (limnetic) habitats in freshwater fish, which has occurred in a variety of fish taxa in temperate as well as tropical environments (Robinson and Wilson 1994; Hulsey et al. 2013; Elmer et al. 2014). Limnetic ecomorphs exhibit characteristic elongated/fusiform body shapes compared to the rather high‐bodied benthic ecomorphs (Webb 1984, 1988). Although fish have diversified along the benthic–limnetic axis in a considerable number of cases, still more commonly fish have not diversified and a single generalist population occupies a lake (Bolnick 2011). Identifying the conditions that have facilitated or hindered population divergence and speciation remains thus a key objective in enhancing our ability to predict evolutionary processes.

Crater lakes in Africa and Nicaragua inhabited by cichlid fishes represent a unique setting to investigate the process of sympatric speciation in a replicated manner and at different stages of the divergence continuum (Elmer et al. 2010b; Kautt et al. 2012; Malinsky et al. 2015). Studying different stages of population divergence, including the earliest stages, holds great promise to identify the relevant mechanisms and conditions driving speciation (Via 2009). Different crater lakes located even within a narrow geographic area are often of different age and usually extremely remote, isolated bodies of water that are typically not connected to other lakes or rivers, making multiple invasions rather unlikely (but see Martin et al. 2015). Thus, in contrast to fish in postglacial lakes in which divergence may have often happened due to character displacement after a secondary invasion (Schluter 1996), divergence in crater lake cichlids is often assumed to have happened in sympatry. This should allow for a more direct study of the conditions that can lead to speciation with gene flow, not contingent or affected by the initial amount of population divergence and reproductive isolation that was already present at the time of secondary contact.

Similarly to fish in postglacial lakes, on the other hand, the evolutionary outcomes of crater lake cichlid populations vary. Haplochromine cichlids in Uganda, for example, have repeatedly evolved an overall more limnetic body shape without diversifying after the colonization of crater lakes (Machado‐Schiaffino et al. 2015). In a small crater lake in Tanzania, cichlids are diverging along the littoral zone and the deeper benthic areas instead of the benthic–limnetic axis (Malinsky et al. 2015).

Midas cichlids (belonging to the *Amphilophus* sp. species complex) that have independently colonized several crater lakes in Nicaragua from the same source population of the great lakes Nicaragua and Managua, but probably at different time points, show a variable pattern (Elmer et al. 2010b): They have independently evolved into one limnetic and several benthic species in the two Crater Lakes Apoyo and Xiloá (Barluenga et al. 2006; Kautt et al. 2012; Elmer et al. 2014), whereas in other crater lakes, they have not diversified along the benthic–limnetic axis (Elmer et al. 2010b). We note that the limnetic niche is completely absent in the relatively shallow (mean depth around 8–12 m) and turbid source lakes (Barlow 1976; Elmer et al. 2010b). One potential explanation is that the variability in body elongation is a key factor that influences the potential for intrapopulation competition and thus frequency‐dependent disruptive selection. Within the repeated adaptive radiations of Midas cichlids (where 13 species have been described so far; Recknagel et al. 2013a, b), this variability seems to be correlated with the mean depth of the respective crater lake (Recknagel et al. 2014).

In this regard, Crater Lake Asososca Managua – the focal lake of this study – has a mean depth (54.3 m) similar to that of Crater Lake Xiloá (60 m). Further, the population in Lake As. Managua exhibits a large variation in body shape elongation, and individual body shapes are correlated with their lower pharyngeal jaw shapes and feeding ecology (Kusche et al. 2014). Thus, one of the conditions (condition 1 outlined above) for divergence along the benthic–limnetic axis seems given. Yet, Crater Lake As. Managua is thought to harbor only a single population of Midas cichlids (Barluenga and Meyer 2010; Kusche et al. 2014). Importantly, however, fish had previously only been collected at the shore and never in the middle of the lake, where limnetic fish are thought to forage. Without a representative sample from both habitats, population genetic methods are arguably underpowered for testing population divergence. Asososca Managua is also the youngest of the crater lakes (max. 1245 years old; Pardo et al. 2008) known to harbor an endemic species of the adaptive radiation of Midas cichlids (Elmer et al. 2010b), *Amphilophus tolteca* (Recknagel et al. 2013b), and the population may thus be at a much earlier stage of divergence than the benthic–limnetic species pairs in Crater Lakes Apoyo and Xiloá. In other words, the propensity to diverge along the benthic–limnetic axis in Midas cichlids seems to be related to the depth of the crater lake, yet the extent of divergence along this axis might depend on the age of the resident population (time since colonization).

Here, we set out to investigate whether the second and third conditions for incipient sympatric speciation as outlined above are present and whether population divergence may currently be happening in the extremely young population of *A. tolteca* in Crater Lake As. Managua. To do so, we caught fish from the open‐water zone in the middle of the lake (limnetic habitat) in addition to the shore (benthic habitat) and used an integrative approach to test whether there is morphological (body elongation), ecological (long‐term diet in form of stable isotope signatures), and genetic (microsatellites) divergence between fish captured in the two different habitats. If the variation in body shape is the result of specialization along the benthic–limnetic axis, we would predict fish that preferentially use the limnetic habitat to exhibit on average a more elongated body shape and limnetic lifestyle (i.e., a higher proportion of carbon from a limnetic source in their diet) compared to individuals from the benthic habitat (condition 2). So far, this prediction has never been tested in Midas cichlids, as sampling of fish usually occurred exclusively close to the shore (i.e., the benthic habitat) and during the breeding season. Differential habitat use, even in a very small lake like As. Managua (ca. 900 m in diameter), is a key factor that may increase the likelihood of population divergence via habitat isolation (Servedio et al. 2011). In fact, habitat isolation is a necessary assumption in a model of sympatric speciation that was specifically tailored to Midas cichlids in Crater Lake Apoyo (Gavrilets et al. 2007). We note that Midas cichlids form seasonally monogamous pairs that breed at the shore, but that pair formation happens probably in the respective habitat. Thus, habitat isolation would act as an effective isolating barrier during the time of pair formation and not breeding (Gavrilets et al. 2007).

Another factor that had so far not been explicitly tested in Midas Cichlids is the role of phenotypic plasticity in body shape elongation. While a previous QTL mapping study in closely related limnetic and benthic species of Midas cichlids in Crater Lake Apoyo already demonstrated a strong genetic component in the determination of body elongation (Franchini et al. 2014), in this study we investigated the contribution of plasticity to the phenotypic variation in body elongation observed in Midas cichlids. In other words, we tested whether differences in the ecologically relevant trait of body elongation are presumably mostly genetically determined (condition 3) or can be attributed to phenotypic plasticity. This question was investigated with split‐brood design experiments conducted in the laboratory in which one half of the fish were reared in tanks with a constant water flow and the other half in control tanks without water flow. In addition to the focal species from As. Managua, we also subjected broods of three other Midas cichlid species (two limnetic species from different crater lakes and one benthic species from the source population of the crater lakes) to the experiment.

Finally, we used a previously in‐house generated RAD‐seq data set from fish captured at the shore to reconstruct the demographic history of the population in Crater Lake As. Managua using coalescent simulations and the site frequency spectrum. This allowed us to put our results of the putative stage of population divergence in perspective to the actual time of colonization and size of the founder population.

## Materials and Methods

### Sampling

**Figure 1:**
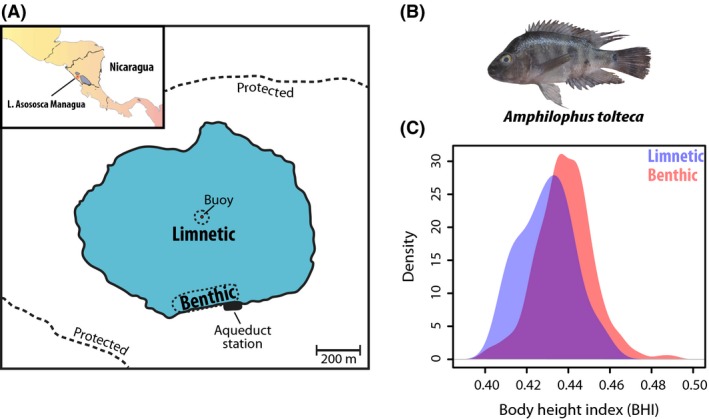
Crater Lake Asososca Managua is located in the center of Nicaragua's capitol, the city of Managua, and protected from its surroundings. (A) Fish were collected in the limnetic (middle of the lake) and benthic (shore) zones of the lake. (B) Representative specimen of the focal species of this study, *Amphilophus tolteca*, endemic to As. Managua. (C) Relative frequency (kernel density estimate) of body height index (i.e., a measure of overall body elongation) of fish collected from the middle of the lake (blue) and the shore (red).

Fish were captured with gill nets or angling in Lake Asososca Managua in 2013. Most of the fish (*n* = 250) were captured at the shore close to the aqueduct main station (Fig. 1A). In addition, the protected environment of Crater Lake As. Managua (surrounded by a fence with only restricted access) allowed us to catch a sufficient number of fish from the middle of the lake (*n* = 27) by attaching gill nets to a buoy; without a fixed anchor point, gill nets get tangled up and leaving nets or a buoy undisturbed in the middle of a lake is not feasible in other crater lakes in Nicaragua. For fish from the middle of the lake and a subset (randomly chosen) of those from the shore (*n* = 27), standardized photographs were taken from the lateral view including a size standard. For these samples, also tissue samples were taken and preserved in pure ethanol for stable isotope and population genetic analyses. An overview about the samples and the morphological, stable isotope, and microsatellite data is provided in Table S1, Supporting information. Samples for RAD sequencing used for demographic inference (*n* = 49) were collected in earlier field expeditions in 2007 and 2010 at the shore and processed in the same way. Sampling was approved and performed according to the regulations of the local authorities, the Ministerio de Ambiente y Recursos Naturales, Nicaragua (MARENA).

### Measurements of body elongation

Overall body elongation in fish is a useful univariate measure to capture the main axis of body shape variation that distinguishes limnetic and benthic individuals (Kusche et al. 2014; Recknagel et al. 2014). The relative extent of body elongation was measured in terms of the body height index (BHI). The body height index is defined as the ratio of body height (distance between insertion of the pelvic fins and the most anterior point of the dorsal fin) to standard length (distance between the snout and the most posterior point of the caudal peduncle). Higher values of the BHI denote thus more high‐bodied (benthic) individuals, whereas lower values of the BHI denote more elongated (limnetic) individuals.

Body height and standard length were initially measured in the field for a large number of individuals captured at the shore (*n* = 250) with calipers. For fish from the middle of the lake (*n* = 26; one individual was missing its pelvic fins and its body height could thus not be measured reliably), the BHI was measured from individual photographs. To rule out that the two different measuring modes (calipers, photographs) had any effect, we remeasured the BHI for a subset of fish from the shore (*n* = 27) – later used for stable isotope and population genetic analyses – from individual photographs. These analyzed specimens from the shore represent a random subsample of the 250 collected individuals. This random selection and the two different measurement modes are unlikely to have biased our results, as the subset was indistinguishable in terms of the BHI from the whole set of fish from the shore (subset: mean = 0.437 ± 0.013 SD; whole set: mean = 0.438 ± 0.014 SD; Welch two‐sample *t*‐test, *t* = 0.443, *P* = 0.660).

### Stable isotopes

We compared carbon and nitrogen stable isotope signature of individuals captured in the limnetic (*n* = 25) and the benthic zones (*n* = 25). Stable isotope analyses were conducted on ethanol‐preserved muscle tissue extracted from dorsal musculature. Tissues were dried at 55°C until there was no more change in mass (ca. 48 h). Muscle samples were subsequently ground up and those between ca. 0.7 and 0.9 mg were placed in tin capsules for analyses. Gas chromatography–combustion–isotope ratio mass spectrometry was performed at the Isotopes Laboratory of the Limnological Institute of the University of Konstanz. Values of *δ* ^13^C ‰ were corrected for lipid content (Kiljunen et al. 2006).

### Phenotypic plasticity

To test whether the observed variation in body shapes could be due to phenotypic plasticity, we performed split‐brood design breeding experiments in the laboratory. Single broods were divided into two groups and raised at approximately equal densities in identical GFK tanks with rounded corners (ca. 2000 L volume, 160 cm diameter) either with a constant circular water flow or in control tanks without water flow (Fig. S1, Supporting information). The flow regimes in these tanks were designed to simulate the increased swimming demand related to foraging in the limnetic habitat compared to the benthic habitat. In addition to *A. tolteca* – the focal species endemic to Crater Lake As. Managua – we included *Amphilophus citrinellus* from the great lakes, which has acted as the source population of all Midas cichlids endemic to the crater lakes and is thought to resemble the ancestral state of the whole species complex. Moreover, we included the two limnetic species of Midas cichlids, *Amphilophus zaliosus* and *Amphilophus sagittae*, endemic to Crater Lakes Apoyo and Xiloá, respectively. Broods of the four species stemmed from stock fish kept in the animal research facility of the University of Konstanz. At the start of the experiment, fish of *A. sagittae* were approximately 5 months (mean weight 2.9 g ± 1.2 g SD), *A. zaliosus* ca. 7 months (6.8 g ± 1.7 g SD), *A. citrinellus* ca. 8 months (8.6 g ± 1.8 g SD), and *A. tolteca* ca. 12 months old (35.4 g ± 17.2 g SD). Samples sizes for control and treatment groups of the four species were 41 and 42 for *A. sagittae*, 16 and 31 for *A. zaliosus*, 24 and 24 for *A. citrinellus*, and 16 and 17 for *A. tolteca*, respectively. In control tanks, water inflow from the filter was directed from the top to the center of the tanks (pump: Eheim type 2260, 65 W power), whereas in treatment tanks, the hose conducting the inflow was aligned with one of the side walls to create a constant circular water flow. This current was further enhanced by two additional water pumps aligned with the filter hose on the same side of the tanks. In control tanks, the two additional pumps were attached to opposite sides of the tanks and directed at the center canceling out any water current. Water current in treatment tanks was measured with an Aquadopp HR‐Profiler (Nortek) and estimated to be on average ca. 14 cm/s across the water column and the whole tank (measured in 2‐min intervals in each of the four corners). The experiment ran for 6 months, after which all fish were photographed. Standard length and body height were taken from the photographs to calculate the BHI. Due to logistical reasons, fish from As. Managua could only be included 2 months after the start of the experiment resulting in a total treatment time of 4 instead of 6 months for this species. Fish were fed without any caloric restrictions and handled according to permit number T‐13/13. All statistical analyses were carried out in R (R Development Core Team 2014).

### Microsatellites

Total genomic DNA was extracted from ca. 1 mm^3^ muscle tissue using a proteinase *K* digestion followed by sodium chloride extraction and ethanol precipitation (Bruford et al. 1998). A total of 52 samples (DNA extraction failed for two samples) from the middle of the lake (*n* = 26) and the shore (*n* = 26) were successfully genotyped at 13 microsatellites loci: Abur28, Abur45, Abur82, Abur151 (Sanetra et al. 2009); burt‐kit (Salzburger et al. 2007); M1M, M2, M7, M12 (Noack et al. 2000); TmoM7 (Zardoya et al. 1996); UNH011, UNH012, UNH013 (Kellogg et al. 1995). All loci were PCR‐amplified with fluorescent reverse or forward primers (HEX, FAM, and NED dyes), and fragment lengths were determined with the internal size standard Genescan‐500 ROX (Applied Biosystems, Waltham, MA) on an ABI 3130 Automated Sequencer (Applied Biosystems, Waltham, MA) with the GeneMapper v4.0 (Applied Biosystems, Waltham, MA) software. Scoring errors, large allele dropout, and null alleles were checked employing the program MICROCHECKER (Van Oosterhout et al. 2004). The most supported number of genetic clusters was determined using the model‐based Bayesian approach of STRUCTURE v2.3 (Pritchard et al. 2000). A burn‐in period of 100,000 steps followed by 500,000 Markov chain Monte Carlo iterations was sufficient to ensure convergence. Five independent runs each assuming between one and three genetic clusters (*K* = 1–3) were performed using the admixture model (each individual draws some fraction of its genome from each of the *K* populations) with correlated allele frequencies. Genetic differentiation by means of the fixation index *F*_ST_ was estimated with ARLEQUIN v.3.5.1.3 (Excoffier et al. 2005; Excoffier and Lischer 2010) using 20,000 permutations to determine statistical significance.

### RAD sequencing

Population genomic data were generated following a double‐digest RAD sequencing approach (Peterson et al. 2012) with minor modifications (Recknagel et al. 2013a). The genomic library (pool of 50 individually barcoded samples) was single‐end sequenced for 101 cycles using an Illumina HiSeq 2000 platform at the genomics core facility of TUFTS University (Boston, MA). After quality inspection (there was no quality drop‐off at the end of the reads, and thus, no trimming was performed), individually barcoded reads were demultiplexed using the STACKS v.1.29 software pipeline, filtering out low‐quality reads (flags: ‐w 0.1 ‐s 25 ‐r ‐c –q) (Catchen et al. 2011, 2013). Reads were then mapped to a reference genome assembly of an individual of *A. citrinellus* from great lake Nicaragua (Elmer et al. 2014) with BWA v.0.7.12 (Li and Durbin 2009). Custom bash scripts were used to remove reads mapping to several positions in the genome, containing soft‐clipped positions, or showing a mapping quality of less than 25. Genotyping was carried out with STACKS using a minimum of five reads for each locus, an upper bound of 0.05 for the error rate and a 5% significance level cutoff. The correction module (rxstacks) was used to correct individual genotype calls and remove loci being confounded in more than 25% of individuals or showing an excess of haplotypes within populations. Individual genotype calls with a log‐likelihood of less than −10 were filtered out and did not contribute to any subsequent analyses. On average, 67,980 ± 13,504 SD loci with a mean coverage of 11.7 ± 1.7 SD reads were obtained per individual. The last two positions of our reads exhibited an excess number of, probably artificial, polymorphisms, and those loci with such polymorphisms were hence excluded (i.e., blacklisted). Furthermore, loci deviating from Hardy–Weinberg equilibrium (HWE) (5% significance level) or containing more than three SNPs were excluded from further analyses. HWE exact tests (Wigginton et al. 2005) were performed in Plink v.1.19beta (Purcell et al. 2007). Only one SNP per RAD‐tag locus was used for all analyses to reduce the effect of nonindependence (linkage) among markers.

The demographic history was inferred performing coalescent simulations in FASTSIMCOAL2 (Excoffier et al. 2013). Loci presumably located in coding regions were identified via a BLASTN search against a compilation of transcriptomic data from various species and tissues of Midas cichlids (Elmer et al. 2010a; Henning et al. 2013; Manousaki et al. 2013) and excluded. The minor joint site frequency spectrum (MSFS) was created as described in Kavembe et al. (2016). Briefly, sample sizes were projected downwards to 25 individuals (50 alleles) using *δ*a*δ*i's projection function (Gutenkunst et al. 2009). The final two‐dimensional SFS was built from an effective sequence length of 3.78 Mb and contained 10,075 polymorphic sites. Inferred parameters were converted into demographic units using a substitution rate of 7.5 × 10^−9^ per site and generation (Guo et al. 2013). For each demographic model, between 50 and 125 independent runs were performed, consisting each of 50 rounds of parameter estimation via the ECM algorithm with a length of 100,000–250,000 coalescent simulations each (increasing by 5000 steps each round). Parametric bootstrapping (*n* = 50) was performed to obtain 95% confidence intervals (Excoffier et al. 2013).

## Results

### Body shape differences

In concordance with our prediction that fish collected in the open‐water area of the lake (limnetic zone) would exhibit on average a more fusiform/elongated body shape than fish caught at the shore (littoral part of the benthic zone), the former were on average more elongated than the latter (Fig. 1C): The average BHI of fish from the middle of the lake was 0.430 ± 0.013 SD compared to an average BHI of 0.438 ± 0.014 SD for fish from the shore (Welch two sample *t*‐test, *t* = 3.304, df = 31.08, *P* = 0.002). This difference is unlikely to be a result of allometric effects (e.g., smaller fish are more elongated) as fish in the two groups did not significantly differ in absolute standard length (Welch two‐sample *t*‐test, *t* = 0.591, df = 35.363, *P* = 0.558) or in absolute body height (Welch two‐sample *t*‐test, *t* = −0.258, df = 33.264, *P* = 0.798).

### Stable isotopes

**Figure 2:**
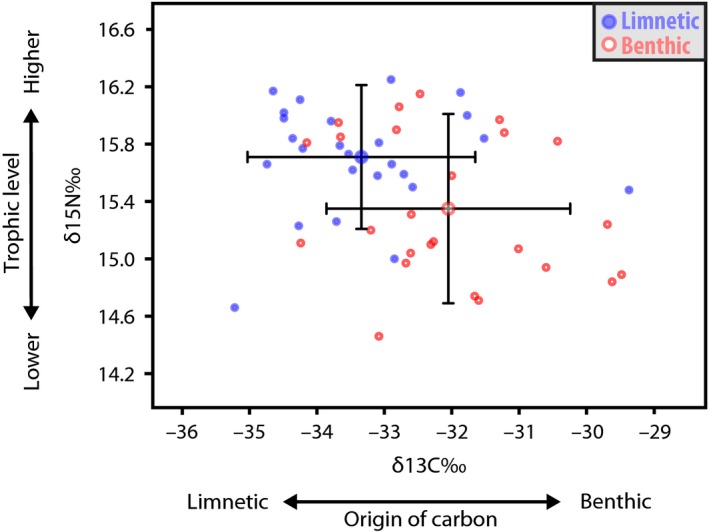
Fish collected from the limnetic zone (blue) exhibit on average higher values of *δ* ^15^N than fish from the benthic zone (red), suggesting a slightly higher trophic position, and lower values of *δ* ^13^C, implying that their source of carbon is of a more limnetic origin. Large dots represent the mean ± SD.

To investigate whether fish captured in the limnetic zone actually exploited this habitat compared to fish captured at the shore that presumably feed predominantly on benthic prey, we performed stable isotope analyses on both groups. Indeed, fish captured in the middle of the lake had significantly different carbon and nitrogen isotopic signatures compared to those collected at the shore (MANOVA Pillai 0.273, approximate *F*_2,47_ = 8.808, *P* = 0.001). This suggests that the long‐term diet of these two groups differs both in the source of carbon (univariate ANOVA, *F*_1,48_ = 11.949, *P* = 0.001) as well as the trophic level as indicated by the nitrogen isotope composition (univariate ANOVA, *F*_1,48_ = 8.230, *P* = 0.006). Fish collected in the limnetic zone of the lake were depleted in ^13^C (mean *δ* ^13^C = −33.34 ± 1.28 SD) compared to those from the benthic zone (mean *δ* ^13^C = −32.05 ± 1.37 SD) (Fig. 2). Values of *δ* ^13^C allow to draw inferences about the relative contribution of different primary producers as a source of carbon of lacustrine animals because planktonic primary producers are depleted in ^13^C compared to benthic primary producers (France 1995a,b; Hecky and Hesslein 1995). Fractionation between consumers and their food is negligible, resulting in *δ* ^13^C values being conserved throughout the food chain (DeNiro and Epstein 1981). The difference in *δ* ^13^C values between primary producers is thus consistently passed on to primary and secondary consumers (Zanden and Rasmussen 1999; Beaudoin et al. 2001; Post 2002). Our data suggest that the diet of fish captured in the limnetic zone of the lake had a higher proportion of carbon of a limnetic origin than the diet of fish sampled in the benthic zone.

Furthermore, fish captured in the limnetic zone were significantly enriched in ^15^N (mean *δ* ^15^N = 15.71 ± 0.38 SD) compared to those captured in the benthic zone (mean *δ* ^15^N = 15.35 ± 0.50 SD) (Fig. 2). Consumers generally become enriched in ^15^N compared to their food source (DeNiro and Epstein 1981; Minagawa and Wada 1984; Post 2002). Hence, *δ* ^15^N values of consumer's tissues serve as an indicator of average trophic position (Zanden and Rasmussen 1999; Post 2002). The difference in the mean value of *δ* ^15^N between fish captured in the middle and the shore of the lake was relatively small with the former being on average ca. 0.5 ‰ higher than the latter. The difference between the two groups could be due to two factors. On the one hand, following the standard interpretation of *δ* ^15^N values, fish from the limnetic zone of the lake might occupy a slightly higher trophic position than those from the benthic zone as they consume on average more secondary consumers. Alternatively, the difference could be due to the fact that the primary producers supporting the food chains of benthic and limnetic fish differ in their *δ* ^15^N values, as planktonic primary producers are slightly enriched in ^15^N compared to benthic primary producers (Zanden and Rasmussen 1999). Given the observed difference in *δ* ^13^C values between the two groups – suggesting a different origin of carbon – this is also a plausible explanation. Either way, overall our data suggest a rather similar position in the trophic chain, yet evidence for clear differences in the diet emerges from the stable isotope data between fish from the limnetic zone of the lake and those from the benthic zone.

### Phenotypic plasticity

**Figure 3:**
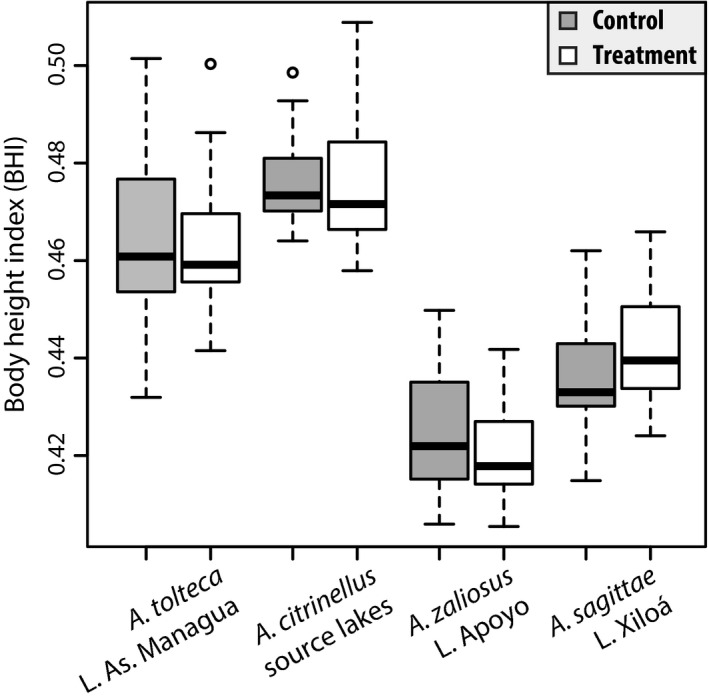
Body height index of control (shaded in gray) and treatment (white) groups of all four species included in the phenotypic plasticity experiment. Only species differ in their body height index (body elongation), whereas treatment did not have a significant effect.

In concordance with our predictions, fish from the benthic and limnetic zones differed significantly in body elongation and long‐term diet. A possible explanation for this result is phenotypic plasticity. Foraging in the open‐water zone could lead to more elongated body shapes due to an increased swimming demand (Johansson and Andersson 2009). Thus, in an attempt to test whether the differences in body shape elongation could be at least partly explained by phenotypic plasticity, that is, whether enduring swimming would lead to more elongated body shapes, we performed experiments in a split‐brood design. Using single broods, in addition to the focal species of this study, *A. tolteca*, we included *A. citrinellus* from the great lakes (the source population of the crater lakes) and the two described limnetic species of Midas cichlids, *A. zaliosus* and *A. sagittae*, from Crater Lakes Apoyo and Xiloá, respectively. After four to six months of treatment (four for *A. tolteca* and six for the other three species), we found that neither treatment nor the interaction of species and treatment had a significant effect on body elongation (two‐way ANOVA, *F* = 1.126, *P* = 0.290; *F* = 1.784, *P* = 0.152), while there were significant differences among the four species (*F* = 205.880, *P* < 2 × 10^−16^). The average body height indices for the control and treatment groups after the experiment were 0.464 ± 0.017 SD and 0.464 ± 0.014 SD for *A. tolteca*, 0.476 ± 0.009 SD and 0.477 ± 0.014 SD for *A. citrinellus*, 0.424 ± 0.012 SD and 0.421 ± 0.009 SD for *A. zaliosus*, and 0.436 ± 0.011 SD and 0.442 ± 0.011 SD for *A. sagittae,* respectively (Fig. 3). The relatively high BHI values of individuals from *A. tolteca* in our plasticity experiment compared to wild‐caught fish are probably due to the fact that we used single broods and the parents of the *A. tolteca* brood were by chance relatively high‐bodied. The differences among species reflect the differences found in nature (Elmer et al. 2010b) and are probably not merely influenced by the slightly different age of the four groups. Thus, plasticity induced by increased swimming demands, as expected for fish inhabiting the limnetic zone, is unlikely to contribute significantly to the large degree of inter‐ and intraspecific variation in body elongation observed in Midas cichlids.

### Genetic differentiation

**Figure 4:**
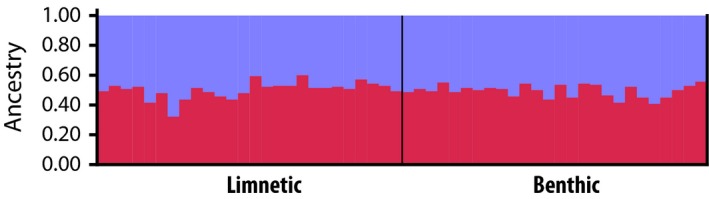
Structure analysis based on 13 microsatellite markers assuming *K* = 2 clusters.

The differences in body shapes and long‐term diet suggested that fish exhibit individual differences in their habitat preference, with some fish predominantly exploiting the limnetic open‐water habitat, while others exploit the benthic shore‐associated habitat. We were interested in whether there is any genetic divergence at neutral markers between the groups. Based on 13 microsatellite markers, we did not find any signs of genetic differentiation or population structuring: The overall *F*_ST_‐value between the two groups was 0.005 (20,000 permutations, *P* = 0.720). Similarly, a STRUCTURE analysis did not reveal more than one genetic cluster. Assuming a priori*,* two clusters (*K* = 2) resulted in admixture proportions around 50% for all individuals (Fig. 4).

### Colonization history

Given the apparent absence of genetic structuring, but the fact that some of the necessary conditions for sympatric speciation are present in the population of Lake As. Managua, we hypothesized that the exceedingly young age of the crater lake population has not been sufficient to build‐up genetic differentiation at neutral markers. Asososca Managua is geologically the youngest crater lake in Nicaragua known to house Midas cichlids (max. age ca. 1245 years; Pardo et al. 2008), yet the exact timing of colonization and the size of its founder population and its current size had not been inferred before. To this end, we used RAD‐seq data to infer the demographic history of the endemic Midas cichlid population of this very small (900 m in diameter) and isolated crater lake. We simulated data in a coalescent framework according to 13 different demographic models (visualized in Fig. S2, Supporting information) and evaluated the fit of the simulations against the empirical data summarized in the MSFS (Table S2, Supporting information) (Excoffier et al. 2013). As a source population, we used *A. citrinellus* from Lake Managua (*n* = 50, see Kautt et al. 2016).

**Figure 5:**
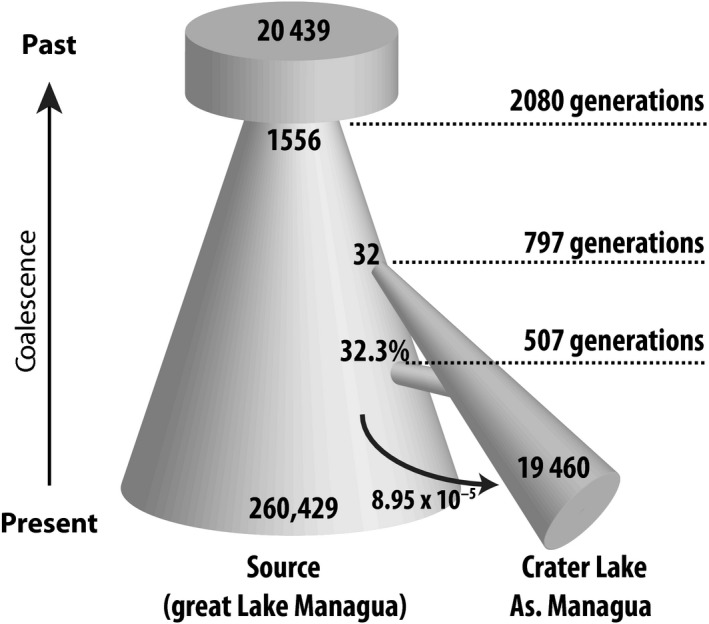
Schematic depiction of the most supported demographic model and the associated maximum‐likelihood point estimates of demographic parameters. Numbers in the model refer to number of diploid individuals, time points refer to number of generations, and the migration rate refers to the probability for an allele to migrate into another deme (in forward time). Note that the model is not drawn in scale, but merely indicates differences in timing and population sizes. In addition, population growth was modeled to be exponential and not linear as shown here.

The most strongly supported model includes a “bottlegrowth” scenario (i.e., a population reduction followed by exponential growth) in the source population, exponential growth in the population of Crater Lake As. Managua after its colonization, and a secondary admixture event as well as continuous migration from the source lake into the crater lake (Fig. 5). According to the maximum‐likelihood point estimates of the most supported model, the source population was reduced from 20,439 (95% CI: 19,289–21,523) individuals to only 1556 (1047–2282) individuals in a bottleneck event 2080 (1498–2885) generations ago, recovering to a current population size of 260,429 (0–712,004) individuals. Note that confidence intervals for population sizes are relatively broad as even slight differences in the estimated exponential growth rates of the parametric bootstrap replicates can translate to large deviations in the population sizes. Crater lake As. Managua was colonized 797 (95% CI: 516–1284) generations ago by only 32 (0–71) individuals. Since then, it has been growing to a current population size of 19,460 (5336–43,039) individuals. In an admixture event that happened 507 (384–652) generations ago, the crater lake population received 32.3% (18.4–50.1%) of its gene pool from the source population (great lake Managua). In addition, since its colonization migration from the source lake (L. Managua) into Crater Lake As. Managua has happened with a probability of 8.95 × 10^−5^ (5.40 × 10^−5^–1.14 × 10^−4^), that is, ca. 9 of 100,000 alleles, per generation. Evidence for an admixture event was unexpected, yet support for admixture events into Crater Lakes Apoyo and Xiloá was found recently (Kautt et al. 2016). Whether the admixture event into Crater Lake As. Managua is real and has facilitated divergence remains to be validated and tested.

In conclusion, Crater Lake As. Managua seems to have been colonized by a small founder population (only around 32 individuals) and very recently (ca. 800 generations ago). Assuming a generation time of 1–2 years (Barluenga and Meyer 2010), the colonization seems to have happened shortly after the formation of the crater lake itself (around 1245 years ago).

## Discussion

Based on the observed correspondence of habitat use with morphology and diet, we can infer that Midas cichlids in Crater Lake Asososca Managua specialize along the benthic–limnetic axis: Fish from the limnetic zone of the lake are on average more elongated than fish from the benthic zone and their diet reflects a more limnetic lifestyle and vice versa. Our laboratory split‐brood design experiments suggest that the differences in body elongation are probably not to a large extent attributable to phenotypic plasticity, but are probably mostly genetically determined. Yet, despite differential habitat use and a presumably strong genetic basis, no population divergence is apparent: Population genetic analyses show support for only one genetic cluster. According to our demographic inferences, the population of Midas cichlids from Lake As. Managua is very young (ca. 800 generations ago) and has been colonized by a very small founder population (about 32 individuals). This suggests that intraspecific competition for resources has probably only begun a few hundred generations ago, which could explain the apparent lack of population divergence uncovered so far at the genomic level.

### Differential habitat use of the benthic and limnetic habitats

Morphological measurements and stable isotope signatures show a match of body elongation, long‐term diet and, importantly, habitat. The match between morphology and stable isotope signature is in agreement with our previous work (Kusche et al. 2014); more elongated fish were depleted in ^13^C and enriched in ^15^N compared to less elongated fish. Yet, we did not recover the whole range of the previously observed variation in *δ* ^13^C and *δ* ^15^N values (Kusche et al. 2014). Our goal was to compare the time‐integrated diet of fish utilizing the limnetic habitat versus those utilizing the benthic habitat, rather than to describe the variation in diet associated with morphology. Therefore, we did not choose the most extreme high‐bodied fish from the shore, but a randomly selected subsample of all the fish captured at the shore. Thus, most of the fish from the shore had an intermediate BHI (subset mean = 0.437). Potentially, we could have recovered a higher range by biasing our sample to include more fish with an extremely high BHI. However, this relation was already clearly established (Kusche et al. 2014). It remained unclear though, if the observed pattern was associated with differences in habitat use *per se*. By sampling Midas cichlids for the first time from the open‐water zone, we provide evidence that the depletion in ^13^C and enrichment in ^15^N of elongated fish compared to less elongated fish is most likely due to the differential use of the limnetic and benthic habitats within the lake. Surely, limnetic and benthic habitats in a lake are not completely discrete and treating them as two distinct habitats is certainly an oversimplification, yet this distinction is a common and reasonable assumption (Bolnick 2011).

While we did not perform a mark–recapture study to test habitat fidelity *per se* (e.g., Bolnick et al. 2009), the stable isotope signatures suggested that fish fed on average consistently, over long periods of time, to a different extent on diets that would be typical of the benthic and limnetic habitats. Therefore, we conclude that individuals preferentially, although probably not exclusively, use either the benthic or the limnetic habitat. Whether the match between eco‐morphological traits is due to genetically based habitat preference or matching habitat choice (*sensu* Edelaar et al. 2008) remains to be tested, but importantly, both processes increase the chance of habitat isolation and thus speciation (Edelaar et al. 2008; Bolnick et al. 2009; Ravigne et al. 2009).

### Differences in body elongation are probably mostly genetically determined rather than plastic

A major question concerning the morphological differences between fish from the limnetic and benthic zones of the lake is whether they could be due to phenotypic plasticity. If phenotypic plasticity was the main factor explaining the differences, population divergence and ultimately speciation may be unlikely (Edelaar et al. 2008; Bolnick 2011; Thibert‐Plante and Hendry 2011). In our phenotypic plasticity experiment, a plastic response was not induced in any of the four tested species: neither *A. citrinellus* from the great lakes (the source population of all crater lakes) that resembles the ancestral state, nor the strongly elongated limnetic species of Midas species *A. zaliosus* (Crater Lake Apoyo) and *A. sagittae* (Crater Lake Xiloá), nor the variable focal species of this study *A. tolteca* exhibited a plastic response in this treatment (Fig. 3). This result stands in contrast to the fact that cichlids exhibit phenotypic plasticity in a number of different traits (Meyer 1987; Kerschbaumer et al. 2011; Machado‐Schiaffino et al. 2014). We note, however, that we only investigated whether treatment had an effect on the elongation of the main body axis as this was the main trait we were interested in. It is thus possible that an undetected plastic response was induced in our experiment (e.g., in other aspects of morphology, behavior, or physiology).

One potential caveat of this experiment is that only one brood per species was tested. Thus, the results cannot be readily generalized to infer the degree of plasticity that may exist within the entire species. Phenotypic plasticity itself may be variable within and among species (Machado‐Schiaffino et al. 2014). However, we believe that the fact that none of the four species showed any pronounced plastic response supports our conclusion. It is also possible that the treatment was applied too late in their ontogeny as earlier developmental stages might be more susceptible to exhibit a plastic response than later stages or that the treatment was not strong enough. Yet, the strength of our treatment was comparable to other studies using a constant water flow in an attempt to induce a plastic response in fish (Peres‐Neto and Magnan 2004; Franssen et al. 2013).

Alternatively, the treatment may have been biologically unrealistic in mimicking the natural conditions. Generally, limnetic fish are thought to exhibit a more fusiform body shape as an adaptation for increased swimming demand (Webb 1984, 1988), yet this is not driven by a constant water current, but rather related to the mode of foraging in the open water. Enclosure experiments in the wild restricting individuals to the shore or the open‐water habitat for foraging would be a more accurate way of testing a role of phenotypic plasticity (Robinson and Parsons 2002), but such experiments have so far not been feasible in Nicaragua for logistical reasons. Nonetheless, whether driven by a different mode of foraging, predator avoidance, or any other potentially unknown reason, the relevant biological stimulus leading to a more elongated body shape is most likely the resulting increased swimming demand. We think our treatment did effectively trigger this stimulus. Thus, we conclude that phenotypic plasticity does not seem to play a major role in explaining the observed morphological differences.

This conclusion is further supported by a study on the genetic basis of the benthic–limnetic morphological divergence of sympatric Midas cichlid species in Crater Lake Apoyo that found that the divergent body shapes are maintained in the laboratory and furthermore identified QTLs that explain some of the morphological differences (Franchini et al. 2014). The significant differences in body shape that we found among the four species in this study (Fig. 3) lend further support to the earlier findings that the differences between species are maintained in captivity even after one to two generations and are most likely to a large extent genetically determined.

### Lack of population differentiation possibly due to very recent origin of intraspecific competition

Population divergence along the benthic–limnetic axis is common in freshwater fish (Robinson and Wilson 1994) and is the basis of sympatric speciation in at least two radiations of Crater Lake Midas cichlids (Barluenga et al. 2006; Elmer et al. 2014). Despite this and the fact that fish in Lake As. Managua differently use the limnetic and benthic habitats, our population genetic microsatellite data suggest that fish from the middle of the lake are not genetically differentiated from those captured at the shore. Note that we are here referring to genetic differentiation at neutral markers and not to relatively restricted highly diverged regions that might differentiate the ecotypes (e.g., Malinsky et al. 2015). This lack of genetic structuring is in agreement with previous investigations based on microsatellite markers that did not find any evidence for more than one genetic cluster in Crater Lake As. Managua (Barluenga and Meyer 2010; Kusche et al. 2014). It is important to note, though, that these studies differed from our approach in that they exclusively used individuals captured at the shore.

Because samples for RAD‐seq were collected exclusively from the shore, we could not use this data to test for genetic differentiation between fish from the limnetic and benthic zones; this part of the project was started before we sampled fish for the first time from the middle of the lake. Nonetheless, as the two groups do not seem to be differentiated at neutral markers (yet), our demographic inferences should not be affected by using only fish from the shore and our estimates of the colonization time and population size therefore unbiased. According to our demographic inferences, Crater Lake As. Managua has been colonized only around 800 (516–1284, 95% CI) generations ago and by a very small founder population consisting of only around 32 (0–71, 95% CI) individuals. The estimate of the current population size of around 19,500 individuals seems biologically reasonable; Midas cichlids are by far the most abundant fish in L. As. Managua (pers. observation) and are today likely at carrying capacity in the relatively small crater lake (900 m in diameter; surface area of 0.74 km^2^). Yet, right after colonization of the new underexploited environment of the crater lake, the small founder population will almost certainly not have been limited by resources.

Generally, selection pressures in the beginning will most likely have been directed at better adapting the founder population as a whole to the crater lake environment in general: Crater lakes are very deep and their water is usually very clear in contrast to the great lakes (the source) that are relatively shallow (mean depth of 8–12 m) and turbid (Barlow 1976; Elmer et al. 2010b). In this regard, it is interesting that *A. tolteca* has evolved a distinct morphology from the source population (Recknagel et al. 2013b) in only 800 generations. It is possible that the founder effect that we have identified here has facilitated the rapid divergence of the crater lake population as a whole in allopatry (e.g., Kolbe et al. 2012), yet population divergence after the colonization of a new environment due to selection can commence extremely fast (Lescak et al. 2015).

In any case, only with time will the population have become large enough for intraspecific competition for resources to elicit frequency‐dependent disruptive selection. Resource limitation due to a high population density is a necessary condition for stable disruptive selection (Bolnick 2011). Hence, it seems possible that the processes of disruptive selection and assortative mating have been at work in *A. tolteca*, but that genetic differentiation has not built up at neutral markers across the genome yet. Neutral genetic differentiation may not be expected in the earliest stages of divergence (Elmer et al. 2010c; Colborne et al. 2016). It seems likely that only few regions in the genome, that is, small genomic islands, that harbor the genetic basis for the observed differences in morphology and trophic ecology are differentiated between the benthic and limnetic ecomorphs in Crater Lake As. Managua, as has been found recently between littoral and benthic ecomorphs of crater lake cichlids in Tanzania (Malinsky et al. 2015) or carrion and hooded crows in Europe (Poelstra et al. 2014). Note that our RAD‐seq data set did not explicitly include individuals from the two habitats and we could thus not perform outlier tests for signatures of selection.

Altogether, it seems that the population in Lake As. Managua is at the earliest stages of population divergence, and we propose that the very young age of the population and the even later onset of disruptive selection have not allowed for enough time to build up genetic differentiation throughout the genome. Alternatively, the population may be stalled in its divergence, and the speciation process may never be completed (Matessi et al. 2001; Nosil et al. 2009b).

### Midas cichlids in Crater Lake As. Managua: a case for incipient sympatric speciation?

Whether population divergence will proceed and ultimately lead to speciation depends foremost on the strengths of disruptive selection and assortative mating (Gavrilets 2005; Bolnick 2011). Determining the strength of selection acting on the benthic–limnetic divergence has so far proofed not feasible in Midas cichlids due to the difficulty of performing experiments in the field (e.g. placing enclosures in the middle of a crater lake) or realistically resembling the open‐water niche of a crater lake (up to 200 m deep) in the laboratory. Yet, the fact that sympatric speciation along the benthic–limnetic axis has happened in two other crater lakes (Elmer et al. 2014) suggests that selection pressures along the benthic–limnetic axis in Nicaraguan crater lakes have been at least in some cases sufficient in driving sympatric speciation in Midas cichlids. However, the specifics matter and every lake environment and every population's demographic history will result in different conditions that may or may not be conducive to sympatric speciation (Bolnick 2011; Martin 2013).

While our results suggest that fish in Crater Lake As. Managua differentially use the benthic and limnetic habitats, it is currently unclear if this would readily translate to reproductive isolation by habitat isolation like in phytophagous insects, for example (Rice 1987; Feder 1998; Via 1999). Midas cichlids form seasonally monogamous pairs that breed at the shore (Barlow 1992), and hence, the spatial segregation breaks down during the time of breeding (Baylis 1976). Yet, if pair formation happened in the respective habitat before pairs move to the shore to breed, differential habitat use would effectively result in assortative mating by habitat (Gavrilets et al. 2007). Behavioral experiments have shown that sympatric benthic and limnetic species from Crater Lakes Apoyo and Xiloá mate assortatively even under laboratory conditions (i.e., in the absence of different habitats), but that pair formation happens before the pairs establish territories for breeding (Baylis 1976; Kautt et al., unpublished data). Hence, active mate preference seems to be a strong mechanism leading to assortative mating in these species, but this does not negate the possibility that habitat isolation is still contributing to reproductive isolation or has played a role in the initial divergence of limnetic and benthic species in these two crater lakes. Different reproductive barriers often act in concert, and their contribution to overall reproductive isolation can change over time (Nosil and Schluter 2011; Nosil 2012). Whether, and to what extent, assortative mating due to habitat isolation or mate preference exists in *A. tolteca* remains to be tested, but our study shows that the conditions for assortative mating by habitat isolation can exist even in a very small crater lake such as As. Managua.

## Conclusions

Divergence along the benthic–limnetic axis is a common theme in the diversification of freshwater fishes (Schluter and McPhail 1992; Robinson and Wilson 1994; Hulsey et al. 2013; Colborne et al. 2016), and in at least two crater lakes in Nicaragua, Midas cichlid fish have speciated along this axis in sympatry (Elmer et al. 2014; Kautt et al. 2016). Theory predicts that disruptive selection due to intraspecific competition for resources and assortative mating due to habitat isolation are necessary conditions for this process (Gavrilets et al. 2007). However, this prediction has never been tested before in Midas cichlids. In complement to previous studies that have focused on the benthic and limnetic species in Crater Lakes Apoyo and Xiloá (Barluenga et al. 2006; Kautt et al. 2012; Elmer et al. 2014), in this study we investigated a much earlier stage of the divergence continuum: population divergence along the benthic–limnetic axis in a species of Midas cichlids, *A. tolteca* (Recknagel et al. 2013b), endemic to the extremely young and small Crater Lake As. Managua. More specifically, we studied whether some of the necessary conditions for sympatric speciation due to intraspecific competition for resources and habitat isolation are given in Lake As. Managua.

In agreement with the prediction of a match between phenotype and habitat, we found that individuals caught in the limnetic habitat are more elongated than fish collected in the benthic habitat. Stable isotope analyses – by integrating diet over longer time spans – further confirmed that fish from the limnetic habitat also exhibit a more limnetic lifestyle. Thus, we conclude that individuals differentially use the two habitats. Together with previous evidence (Franchini et al. 2014), our experiments conducted in the laboratory suggest that the differences we found in the most relevant ecological trait, body shape elongation, are unlikely to be due to phenotypic plasticity and are probably strongly genetically determined. Altogether, our data therefore support the notion that some of the necessary conditions for sympatric speciation are present in Midas cichlids in Crater Lake As. Managua: Individuals vary in genetically determined ecomorphological traits and differentially use the benthic and limnetic habitats. Whether these conditions are *sufficient* for sympatric speciation to occur remains to be tested (e.g., Bolnick 2011; Martin 2013). Here, we did not find evidence for neutral genetic divergence in the population. This could either mean that the strengths of disruptive selection and assortative mating are not strong enough and that the population is therefore stalled in its divergence (Matessi et al. 2001) or that we are dealing with a very recent population divergence (Elmer et al. 2010c; Colborne et al. 2016). While ecological experiments are needed to test the former explanation, our demographic inferences suggest that it is plausible that the apparent lack of genetic divergence is due to a lack of time. In conclusion, our study shows how knowledge about the demographic history can inform on studies of speciation and that some of the necessary conditions for sympatric speciation do occur in nature and can occur even in such a small environment as Crater Lake Asososca Managua.

## Data Accessibility

Morphometric measurements, stable isotope data, and microsatellite genotypes are provided in Table S1, Supporting information. The variant call format (VCF) of the RAD‐seq data is available as Data set S1, Supporting information.

## Conflict of Interest

None declared.

## Supporting information


**Figure S1.** Experimental setup of the phenotypic plasticity experiment.

**Figure S2.** Schematic illustration of all 13 tested demographic models.

**Table S1.** Morphometric measurements, stable isotope data, and microsatellite genotypes.

**Table S2.** Support for tested demographic models.

**Data set S1.** RADseq genotype data in variant call format.
