# Supplementary figures and images for: Incipient sympatric speciation in Midas cichlid fish from the youngest and one of the smallest crater lakes in Nicaragua due to differential use of the benthic and limnetic habitats?
Source: Ecol Evol. 2016 Jul 1;6(15):5342–57. doi: 10.1002/ece3.2287 (PMC4984508; doi:10.1002/ece3.2287)

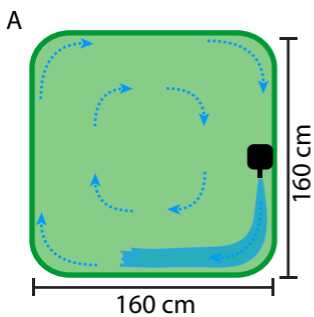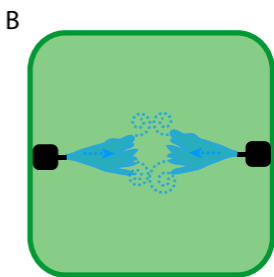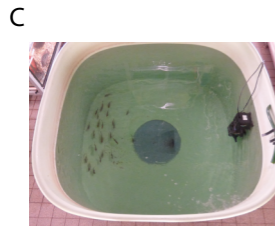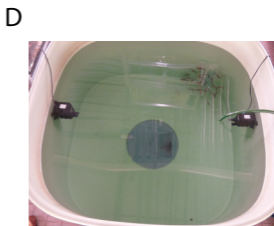

Supplement: Supplementary file 1 — Figure S1. Experimental setup of the phenotypic plasticity experiment. [file ECE3-6-5342-s001.pdf]
