# Supplementary material for: Incipient sympatric speciation in Midas cichlid fish from the youngest and one of the smallest crater lakes in Nicaragua due to differential use of the benthic and limnetic habitats?
Source: Ecol Evol. 2016 Jul 1;6(15):5342–57. doi: 10.1002/ece3.2287 (PMC4984508; doi:10.1002/ece3.2287)
Supplement: Supplementary file 2 — Figure S2. Schematic illustration of all 13 tested demographic models. [file ECE3-6-5342-s002.pdf]

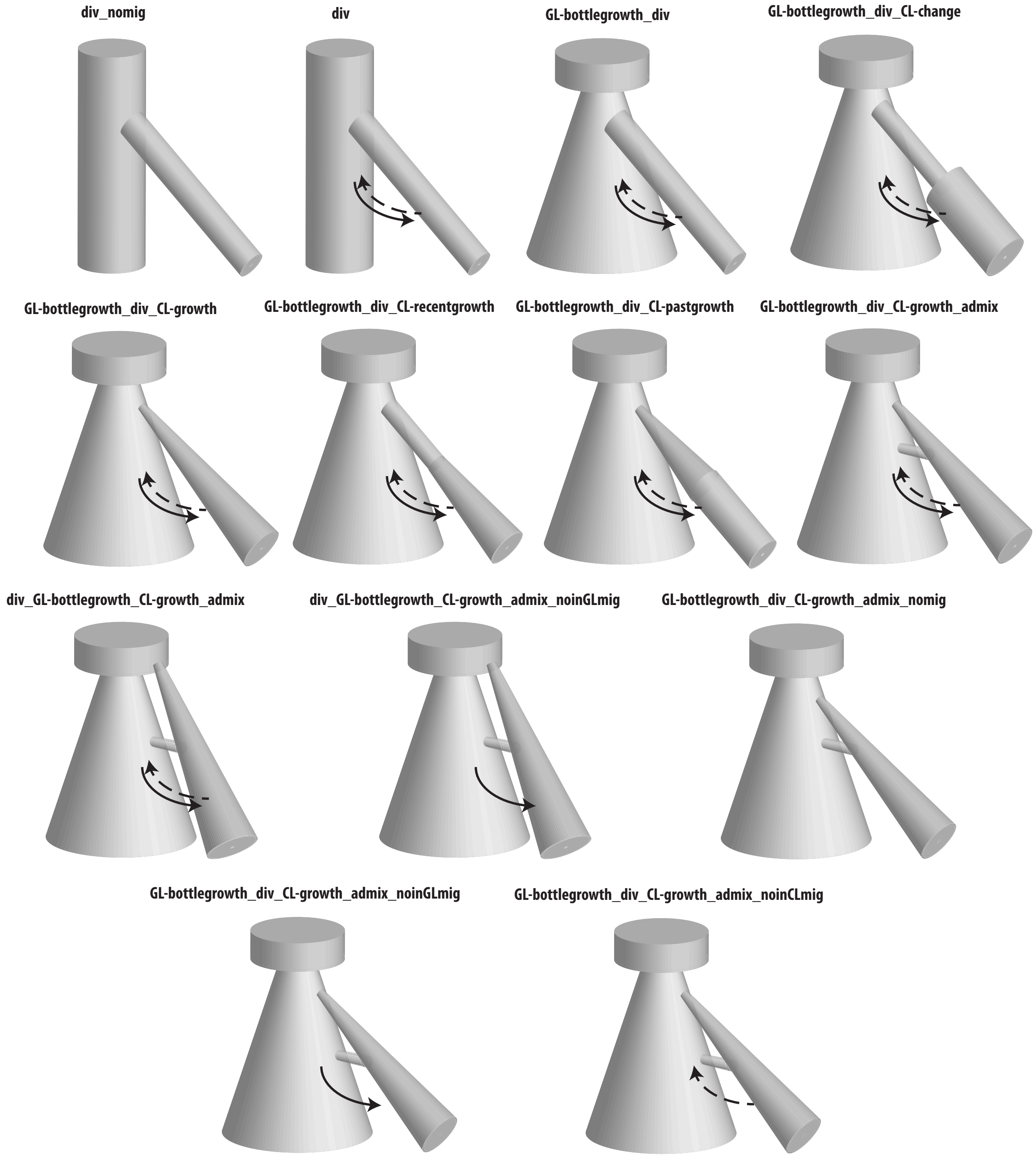
div_nomig
div
GL-bottlegrowth_div
GL-bottlegrowth_div_CL-change
GL-bottlegrowth_div_CL-growth
GL-bottlegrowth_div_CL-recentgrowth
GL-bottlegrowth_div_CL-pastgrowth
GL-bottlegrowth_div_CL-growth_admix
div_GL-bottlegrowth_CL-growth_admix
div_GL-bottlegrowth_CL-growth_admix_noinGLmig
GL-bottlegrowth_div_CL-growth_admix_nomig
GL-bottlegrowth_div_CL-growth_admix_noinGLmig
GL-bottlegrowth_div_CL-growth_admix_noinCLmig
